# Phosphorylation-dependent deubiquitinase OTUD3 regulates YY1 stability and promotes colorectal cancer progression

**DOI:** 10.1038/s41419-024-06526-8

**Published:** 2024-02-13

**Authors:** Liang Wu, Zili Zhou, Yang Yu, Can Cheng, Shuai Zhou, Yuan Yan, Bofan Yu, Yuwei Zhang, Zhengyi Liu

**Affiliations:** 1 Department of General Surgery, The First Affiliated Hospital of University of Science and Technology of China, Division of Life Sciences and Medicine, University of Science and Technology of China, Heifei, 230001 China; 2 Department of Gastrointestinal Surgery, Sichuan Provincial People’s Hospital, University of Electronic Science and Technology of China, Chengdu, 610000 Sichuan China; 3 Department of Breast Surgery, Henan Provincial People’s Hospital, People’s Hospital of Zhengzhou University, People’s Hospital of Henan University, Zhengzhou, 450000 Henan China; 4 Microbiome Laboratory, People’s Hospital of Zhengzhou University, Zhengzhou, 450000 China; 5 Translational Research Institute, Henan Provincial and Zhengzhou City Key Laboratory of Non-coding RNA and Cancer Metabolism, Henan International Join Laboratory of Non-coding RNA and Metabolism in Cancer, Henan Provincial People’s Hospital, Academy of Medical Sciences, Zhengzhou University, Zhengzhou, 450000 Henan China; 6 Key Laboratory of Stem Cell Differentiation & Modification, School of Clinical Medicine, Henan University, Zhengzhou, 450000 China

**Keywords:** Colorectal cancer, Colorectal cancer

## Abstract

Yin Yang 1 (YY1) is a key transcription factor that has been implicated in the development of several malignancies. The stability of YY1 is regulated by the ubiquitin-proteasome system. The role of deubiquitinases (DUBs) and their impact on YY1 remain to be fully elucidated. In this study, we screened for ubiquitin-specific proteases that interact with YY1, and identified OTUD3 as a DUB for YY1. Over-expressed OTUD3 inhibited YY1 degradation, thereby increasing YY1 protein levels, whereas OTUD3 knockdown or knockout promoted YY1 degradation, thereby decreasing the proliferation of colorectal cancer (CRC). Furthermore, PLK1 mediates OTUD3 S326 phosphorylation, which further enhances OTUD3 binding and deubiquitination of YY1. In CRC tissues, elevated the expression level of OTUD3 and YY1 were significantly associated with poor prognostic outcomes. These findings suggest that the OTUD3-YY1 pathway has therapeutic potential in CRC, and OTUD3 plays a critical role in regulating YY1.

## Introduction

CRC is a common malignant tumor of the digestive system, and its morbidity and mortality rank third among malignant tumors [1, 2]. Advances in the development of early screening methods and treatment strategies have led to the improvement of the overall survival rate of CRC patients to some extent [3]. However, due to early metastasis and rapid progression in the later stages, the overall efficacy of the current treatment methods is still poor. Therefore, the survival rate and overall prognostic outcomes are not optimal, which makes CRC one of the most significant medical burdens [4, 5]. Elucidating the specific mechanisms underlying the occurrence and progression of CRC is crucial to improving early detection and reducing mortality.

YY1 is a zinc finger protein that plays a crucial role in regulating the transcription of genes in various cell processes, such as apoptosis, chromatin remodeling, and cell proliferation [6, 7]. Physiologically, YY1 is over-expressed in several malignancies, including metastatic breast cancer [8], colon cancer [9], gastric cancer [10], and prostate cancer [11]. By promoting GLUT3 transcriptional activities, YY1 enhances the Warburg effect and tumor cell proliferation [12]. In Hepatocellular carcinoma, YY1 positively up-regulates EGFR, which enhances tumor cell proliferation and sorafenib resistance [13]. Mechanistically, YY1 directly enhances the ability of Hdm2 to ubiquitinate p53 by facilitating Hdm2-p53 interactions, thereby contributing to tumorigenesis [14]. In CRC, YY1 suppresses the transcription of the tumor suppressor (miR-500a-5p) by binding its promoter, leading to CRC progression [15]. YY1 transcriptionally facilitates CTNNB1 expression and promotes CRC metastasis [16]. Hence, investigating the specific mechanisms of YY1 in CRC development may offer novel insights for the diagnosis and treatment of this disease. Post-translational modification of proteins mainly includes ubiquitination, phosphorylation, methylation, glutamate and other small molecule modifications. Ubiquitination is a more diverse and orderly modification mode of proteins that exerts important effects on numerous cell activities, including body growth, development, gene transcription, cell differentiation, apoptosis and other activities. The ubiquitin-proteasome system is an important regulatory mechanism for intracellular protein degradation [17–20]. Recent studies have demonstrated that Smurf2 modulates YY1 ubiquitination and degradation, thereby inhibiting B cell proliferation and lymphoma occurrence [21]. Like other post-translational modifications, the UB chain can be removed by DUBs. About 100 DUBs have been identified in humans, which can be divided into six signatures: USPs, OTUs, JAMMs, MJDs, UCHs, and MCPIPs [22]. DUBs selectively degrade or stabilize oncogenes, tumor suppressor genes, and cell biology-related proteins that are closely associated with tumorigenesis and tumor progression [17–19]. In the CRC regulation process, DUBs promote tumor progression by up-regulating the expression of key oncogenes [23–26]. For instance, DUB3 significantly promotes NRF2 stability by suppressing K48 ubiquitination, leading to chemoresistance in CRC [23]. Similarly, USP21 promote NSCLC cell proliferation and metastasis by stabilizing YY1 via enhancing its deubiquitination [27]. Despite USP21 depletion, YY1 protein stability remained relatively high, indicating that other DUBs might also contribute to YY1 protein stabilization. In this study, we performed Co-IP, LC-MS, as well as ubiquitin modification omics and identified the ovarian tumor protease (OTU) family member (OTUD3) as a potent DUB for YY1 de-polyubiquitylation. Even though OTUD3 acts as an oncogene in small cell lung cancer and hepatocellular cancer, its role in CRC has not been reported. Therefore, we further explored whether it regulates CRC progression by regulating YY1.

PLK1 is a member of Polo-like kinase family with serine/threonine protein kinase activities. It consists of a Polo box domain (PBD) at the C-terminus, a kinase domain (KD) at the N-terminus, and an intermediate linkage. As a unique functional domain of PLKs, PBD can bind with the substrate [28, 29]. Physiologically, PLK1 plays a vital role in DNA synthesis, damage and repair, as well as in regulating the tumor suppressor gene (P53), implying that it affects all tumor cell cycle aspects [30–32]. We found that PLK1 mediates OTUD3 phosphorylation to enhance its binding abilities, resulting in YY1 stability.

In this study, OTUD3 was identified to be an effective DUB for de-polyubiquitination of YY1. Over-expression of OTUD3 suppressed YY1 degradation and promoted CRC cell growth. Furthermore, phosphorylation of Ser-326 significantly enhanced the effects of OTUD3 in stabilizing YY1. These findings suggest that OTUD3 is a novel DUB for YY1 and regulates its stability in CRC.

## Results

### OTUD3 interacts with YY1

**Fig. 1:**
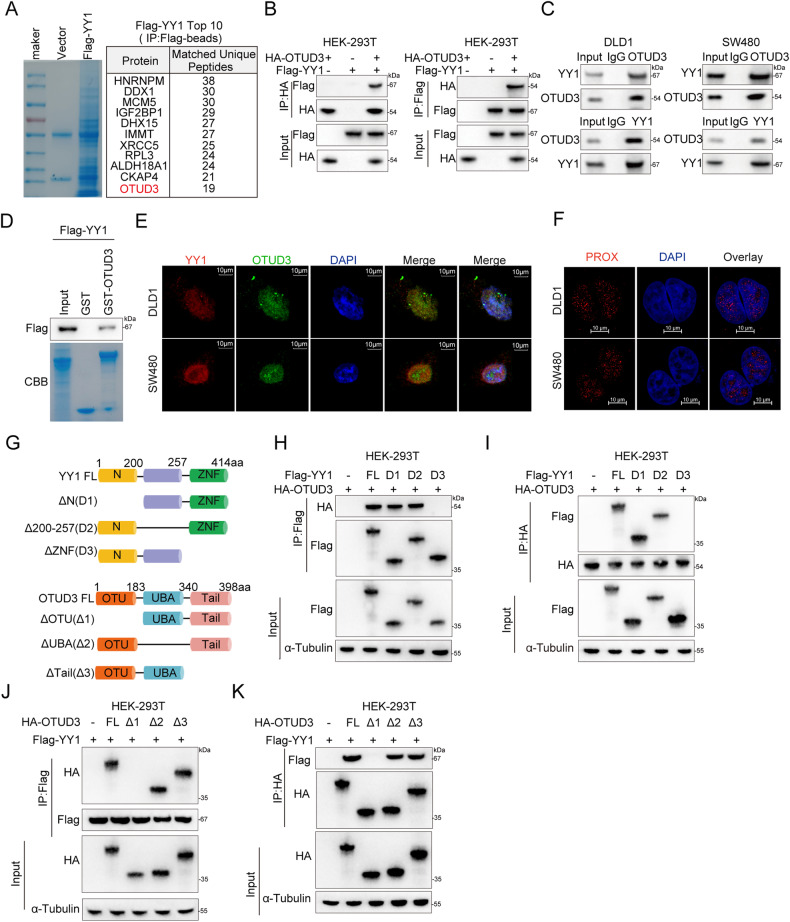
OTUD3 interacts with YY1. **A** Flag-YY1 was transfected in SW480 cells and the anti-Flag antibodies were used for immunocoprecipitation. The coimmunoprecipitated protein was stained by coomassie blue and analyzed by mass spectrometry. **B** HA-OTUD3 and Flag-YY1 were co-transfected into HEK-293T cells. Co-IP was performed to demonstrate the interactions between exogenous OTUD3 and YY1. **C** Co-IP revealed a reciprocal interactions between endogenous OTUD3 and YY1 in SW480 and DLD1 cells. **D** The GST pull-down assay revealed direct interactions between OTUD3 and YY1. Purified Flag-YY1 from HEK-293T cells and incubated with GST protein or GST-OTUD3 protein overnight at 4 °C. GST-OTUD3 complexes were pulled down by Glutathione-Sepharose 4B beads and subsequently detected by immunoblotting with an anti-Flag antibody. **E** Confocal microscopy was used to analyze SW480 and DLD1 cells for the presence of OTUD3 (green) and YY1 (red) antibodies, with the nuclei stained with DAPI (blue). Scale bar, 10 μm. **F** Interactions between endogenous OTUD3 and YY1 in SW480 and DLD1 cells were detected via a proximity ligation assay (PLA). The nuclei were located by DAPI staining, and the red color represents the binding sites of OTUD3 and YY1. Scale bar, 10 μm. **G** Diagrammatic presentation of OTUD3 and YY1 protein full length and various deletion mutants. **H**, **I** Co-IP assays were performed using HA-OTUD3 and Flag-YY1 or its deletion mutants with an anti-HA or anti-Flag antibody. **J**, **K** Co-IP assays were also performed using Flag-YY1 and HA-OTUD3 or its deletion mutant with an anti-HA or anti-Flag antibody.

To elucidate the biological functions of YY1 and its roles in CRC development, Flag-YY1 was over-expressed in SW480 cells. The YY1-associated protein complex in these cells was isolated through mass spectrometry analysis after tandem affinity purification. Coincidentally, DUB OTUD3 was identified on the prey list (Fig. 1A). Notably, OTUD3 was expressed in various CRC cells including SW480, LoVo, DLD1, HT29, and SW620 (Supplementary Fig. S1A). Consistent with the results of the mass spectrometry analysis, coimmunoprecipitation assays (Co-IP) showed that ectopically expressed HA-tagged OTUD3 was detected in Flag-tagged YY1, and vice versa (Fig. 1B). Importantly, interactions between endogenous YY1 and OTUD3 were observed in SW480, LoVo and DLD1 cells (Fig. 1C and Supplementary Fig. S1B). Furthermore, in vitro pull-down assays with purified recombinant proteins showed that YY1 directly bound GST-OTUD3, not GST control (Fig. 1D). Immunofluorescence staining confirmed that YY1 and OTUD3 are predominantly colocalized in the nucleus of CRC cells (Fig. 1E and Supplementary Fig. S1C). An in-situ proximity ligation assay (PLA) was performed to identify the interactions between endogenous YY1 and OTUD3 in CRC cells. The interactions were shown to mainly occur in the nucleus of CRC cells (Fig. 1F and Supplementary Fig. S1D). Mapping the YY1 region according to its functional features, three truncated mutants (ΔN, Δ200-257 and ΔZNF) were constructed. The Co-IP assays revealed that the ZNF region of YY1 is critical for interactions with OTUD3 (Fig. 1G–I). Similarly, the OTU region of OTUD3 mediated the physical interactions with YY1 (Fig. 1J, K). Therefore, the above data demonstrated that OTUD3 is a bona fide YY1-interacting protein.

### OTUD3 maintains YY1 stability

**Fig. 2:**
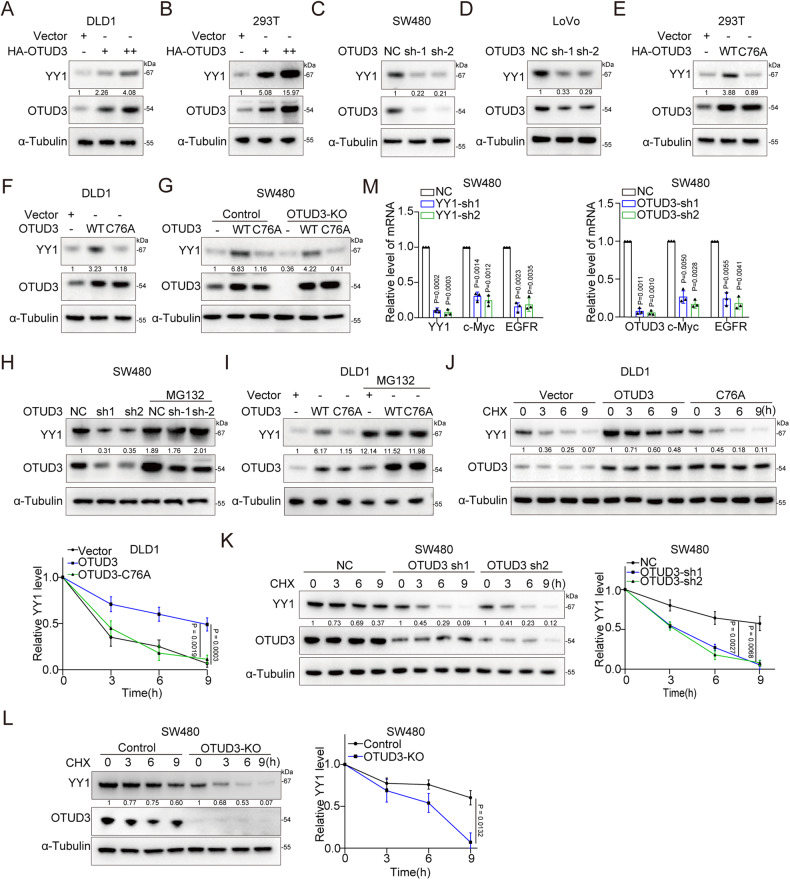
OTUD3 regulates YY1 stability. **A** Western blot was performed to detect YY1 expression in DLD1 cells that had been transfected with increasing amounts of HA-OTUD3. “+” represents transfection of 1 μg HA-OTUD3 plasmids and “++” represents transfection of 2 μg plasmids. **B** Western blot was performed to quantify YY1 expression in HEK-293T cells transfected with increasing concentration of HA-OTUD3. “+” represents transfection of 1 μg HA-OTUD3 plasmids and “++” represents the transfection of 2 μg plasmids. **C**, **D** OTUD3 was silenced by shRNA in SW480 and LoVo cells, and the YY1 protein levels determined by Western blot. **E** HA-OTUD3 (WT) and HA-OTUD3 (C76A) were transfected into HEK-293T cells and YY1 protein expression determined by Western blot. **F** HA-OTUD3 (WT) and HA-OTUD3 (C76A) were transfected into DLD1 cells and YY1 protein expression measured by Western blot. **G** OTUD3 was knocked out by sgRNA in SW480 cells, after which HA-OTUD3 (wild type) or HA-OTUD3 (C76A) were transfected into SW480 cells, and the YY1 protein levels measured by Western blot. (**H**) Western blotting showed that the proteasome inhibitor, MG132, reversed YY1 protein levels in OTUD3 knockdown SW480 cells. **I** HA-OTUD3 (WT) and HA-OTUD3 (C76A) were transfected into DLD1 cells with DMSO or MG132 (10 μM) treatment for 6 h. OTUD3 and YY1 protein levels were examined by Western blot. **J** HA-OTUD3 (WT) and HA-OTUD3 (C76A) were transfected into DLD1 cells with cycloheximide (CHX, 50 μg/ml) treatment for indicated times. OTUD3 and YY1 protein levels were determined by Western blot. **K** OTUD3 was knocked down by shRNA in SW480 cells with CHX (50 μg/ml) treatment for indicated times. OTUD3 and YY1 protein levels were determined by Western blot. **L** OTUD3 was knocked out by sgRNA in SW480 cells with 50 μg/ml cycloheximide (CHX) treatment for indicated times. OTUD3 and YY1 protein levels were evaluated by Western blot. **M** Expression of YY1 or OTUD3 was silenced by shRNA in SW480 cells. Levels of c-Myc and EGFR were determined by qRT-PCR.

To investigate the regulatory effects of OTUD3 on YY1, we over-expressed OTUD3 in DLD1 and HEK-293T cells. The YY1 protein levels were shown to increase in a dose-dependent manner (Fig. 2A, B). Inhibition of OTUD3 expression in SW480 and LoVo cells resulted in suppressed YY1 protein levels (Fig. 2C, D). We then examined whether the deubiquitinating enzymatic activities of OTUD3 are necessary for its effect on YY1. Ectopic expression of wild-type OTUD3, instead of the catalytically inactive mutant OTUD3-C76A, increased YY1 protein levels (Fig. 2E, F). Similarly, in OTUD3 knockout SW480 cells (generated using CRISPR/Cas9), ectopic expression of wild-type OTUD3, instead of mutant OTUD3, restored YY1 protein levels (Fig. 2G). Moreover, YY1 mRNA expression was unaffected by OTUD3 expression (Supplementary Fig. S2A). In addition, we discovered that the OTUD3 suppression-induced YY1 protein destabilization was proteasome-dependent and reversed by treatment with the proteasome inhibitor, MG132 (Fig. 2H, I and Supplementary Fig. S2B). To further examine the effects of OTUD3 on YY1 protein stability, cycloheximide (CHX) was used to block protein synthesis. YY1 exhibited a significantly longer half-life in OTUD3 (wild-type) overexpressing DLD1 cells, while OTUD3 (C76A) did not prolong the YY1 half-life (Fig. 2J). Moreover, cells with OTUD3 knockdown and knockout showed rapid YY1 degradation (Fig. 2K, L and Supplementary Fig. S2C). These results suggest that OTUD3 tightly controls YY1 protein stability.

Next, we investigated the downstream effects of OTUD3-mediated YY1 regulation. Knockdown of YY1 in CRC cells significantly downregulated the expression of previously reported YY1 target genes, such as c-Myc and EGFR. Knockdown of OTUD3 decreased the mRNA levels of these genes (Fig. 2M). This suggests that the effects of OTUD3 on YY1 can alter the downstream genes expression. In summary, OTUD3 regulates YY1 protein stability in a proteasome-dependent manner, which affects downstream gene expression.

### OTUD3 deubiquitinates YY1

**Fig. 3:**
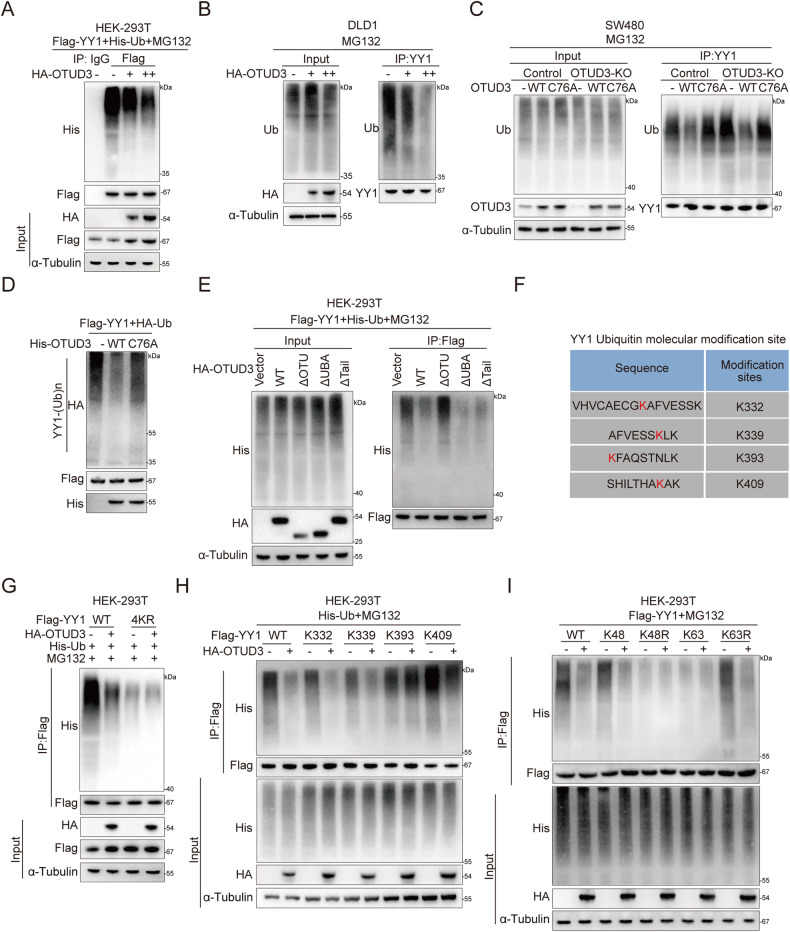
OTUD3 deubiquitinates YY1. **A** Co-IP assays were performed to measure YY1 polyubiquitination in HEK-293T cells that had been transfected with increasing amounts of HA-OTUD3. “+” represents transfection of 1 μg HA-OTUD3 plasmids and “++” represents transfection of 2 μg plasmids. **B** In DLD1 cells. “+” represents the transfection of 1 μg HA-OTUD3 plasmids and “++” represents the transfection of 2 μg plasmids. **C** The expression level of OTUD3 were knocked out by sgRNA in SW480 cells, after which HA-OTUD3 or HA-OTUD3 (C76A) were transfected into SW480 cells. The Co-IP assays were performed to measure the polyubiquitination of YY1 in SW480 cells. **D** An in vitro deubiquitination assay was performed using ubiquitinated Flag-YY1 protein treated with OTUD3-WT or OTUD3-C76A, and the reaction mixture was analyzed by Western blot. **E** HEK-293T cells were co-transfected with plasmids of OTUD3 or OTUD3 deletion mutants and Flag-YY1. Polyubiquitination of YY1 was detected by Co-IP assays. **F** The sequence of YY1 ubiquitination modification sites is provided by ubiquitin modification omics. **G** HA-OTUD3, His-Ub, Flag-YY1 or Flag-YY1 4KR (lys-332, lys-339, lys-393 and lys-409 all mutated to arginine) plasmids were co-transfected into HEK-293T cells. Co-IP assays revealed the polyubiquitination of YY1 in HEK-293T cells. **H** HA-OTUD3, His-Ub, Flag-YY1 or Flag-YY1 mutants (K332, K339, K393 or K409) plasmids were co-transfected into HEK-293T cells. Co-IP revealed the polyubiquitination of YY1 in HEK-293T cells. **I** Flag-YY1, HA-OTUD3, His-Ub or Ub mutants (K48, K48R, K63, K63R) plasmids were co-transfected into HEK-293T cells. Co-IP revealed the polyubiquitination of YY1 in HEK-293T cells.

We then investigated how OTUD3 enhances YY1 stability. Polyubiquitination of endogenous YY1 was detected. Immunoprecipitation of YY1 with an anti-YY1 antibody followed by detection of YY1 polyubiquitination by an anti-ubiquitin antibody revealed that OTUD3 suppression resulted in a sharp increase in expression of the polyubiquitinated form of YY1 protein after MG132 treatment (Supplementary Fig. S3A, B). Conversely, over-expression of wild-type OTUD3 dose-dependently suppressed YY1 polyubiquitination in DLD1 cells and HEK-293T cells (Fig. 3A, B). Polyubiquitination of YY1 was suppressed by over-expression of wild-type OTUD3, instead of the OTUD3-C76A mutant (Supplementary Fig. S3C, D). The additional OTUD3 knockout and rescue assays further confirmed that OTUD3 plays a critical role in regulating YY1 ubiquitination (Fig. 3C). In vitro deubiquitylation assays showed that wild-type OTUD3, but not the OTUD3-C76A mutant, suppressed YY1 ubiquitination (Fig. 3D). Interestingly, OTU domain deletion mutants of OTUD3 lost their ability to regulate YY1 deubiquitination (Fig. 3E).

Modifieromics was performed to elucidate the roles of OTUD3 in YY1 ubiquitination. It was found that OTUD3 significantly reduced the ubiquitination of lys-332, lys-339, and lys-409 of YY1, and slightly up-regulated the ubiquitination of lys-393 (Supplementary Fig. S3E). A YY1-K4R mutant was generated by mutating all the four lysine sites (lys-332, lys-339, lys-393 and lys-409) in YY1. It was revealed that OTUD3 did not deubiquitinate the mutated YY1-K4R (Fig. 3F, G). To identify the specific lysine site responsible for deubiquitination of the YY1 protein by OTUD3, three of the four lysines were mutated to arginine, leaving only one. We found that Lys-332, Lys-339, and Lys-409 are critical sites for OTUD3 to deubiquitinate YY1 (Fig. 3H). To determine which type of YY1 ubiquitination is affected by OTUD3, we co-transfected HA-OTUD3, Flag-YY1 and seven lysine-specific mutants of ubiquitin (K6, K11, K27, K29, K33, K48 and K63) into HEK-293T cells. The OTUD3 was shown to specifically cleave the K48-linked polyubiquitination chains of YY1 protein (Fig. 3I and Supplementary Fig. S3F). In summary, OTUD3 enhances YY1 stability by selectively removing K48-linked polyubiquitination chains from the YY1 protein.

### OTUD3 accelerates CRC progression by up-regulating YY1

**Fig. 4:**
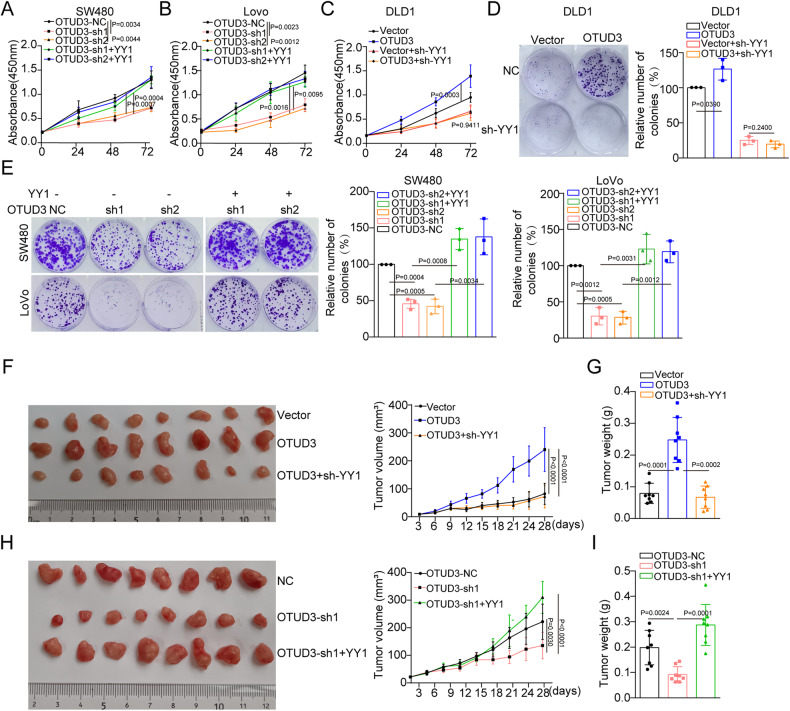
OTUD3 promotes CRC proliferation by up-regulating YY1. **A**–**E** Cell proliferation levels were analyzed in CRC cells transfected with the indicated plasmids and lentiviruses using the CCK-8 and clone formation assays. **F–I** In vivo CRC proliferation was measured by assessing subcutaneous tumor formation (*n* = 8).

To determine whether OTUD3 promotes tumor progression by regulating YY1, we restored the expression of YY1 in endogenous OTUD3 depletion CRC cells or knocked down endogenous YY1 in OTUD3-overexpressing CRC cells. Restoration of YY1 expression weakened the OTUD3 knockdown-induced suppression of CRC proliferation (Fig. 4A, B). Conversely, depletion of endogenous YY1 suppressed the OTUD3-induced CRC proliferation (Fig. 4C). The clone formation assay showed that OTUD3 promoted clone formation by up-regulating YY1 (Fig. 4D, E). Xenograft assays revealed that overexpressed YY1 mitigated the OTUD3 depletion-induced inhibition of xenograft growth, while depletion of endogenous YY1 blocked the OTUD3 overexpression-induced xenograft growth (Fig. 4F–I). Our results confirmed that OTUD3 facilitated tumor growth by up-regulating YY1.

### PLK1 mediated Ser-326 phosphorylation significantly enhances OTUD3 functions on YY1

**Fig. 5:**
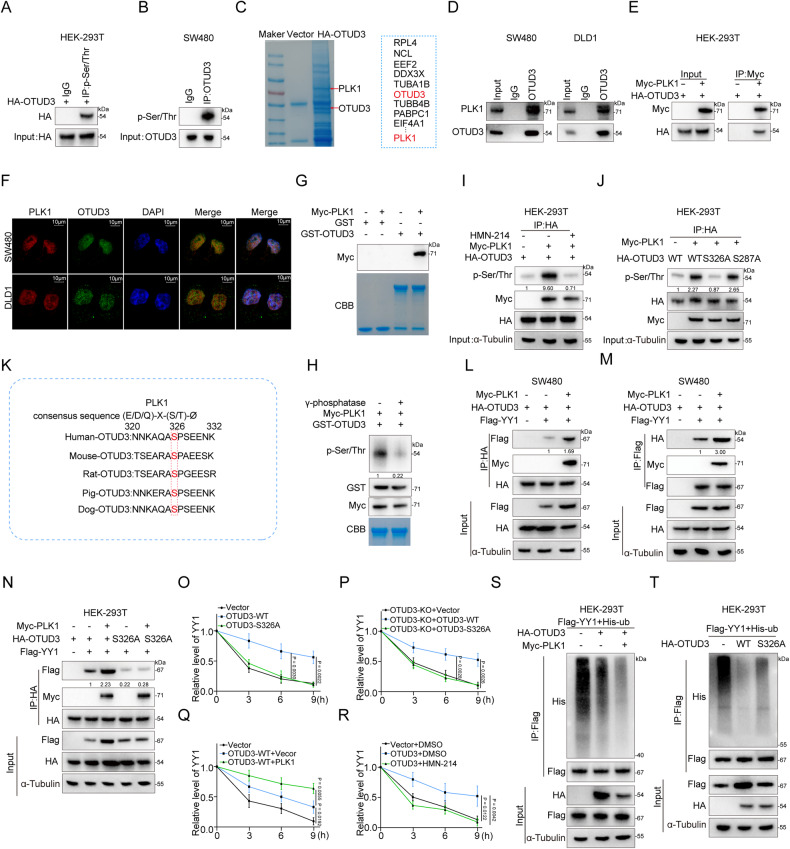
Phosphorylation of OTUD3 mediated by PLK1 enhances its functions on YY1. **A** HA-OTUD3 was transfected into HEK-293T cells and Co-IP assays performed with antibodies against p-ser/thr. **B** Co-IP assays were performed in SW480 with antibodies against OTUD3. The levels of phosphorylated serine and threonine were detected by Western blot. **C** HA-OTUD3 was transfected into DLD1 cells and the anti-HA antibodies were used for immunocoprecipitation. The coimmunoprecipitated protein was stained by coomassie blue and analyzed by mass spectrometry. **D** Interactions between OTUD3 and PLK1 in SW480 cells and DLD1 cells were confirmed by Co-IP assays. **E** Interactions between OTUD3 and PLK1 in HEK-293T cells co-transfected with HA-OTUD3 and Myc-PLK1 were confirmed by Co-IP assays. **F** Location of OTUD3 (green) and PLK1 (red) in SW480 cells and DLD1 cells were detected by immunofluorescence. The nucleus was labeled with DAPI (blue) staining. Cells were analyzed by confocal microscopy. Scale bar, 10 μm. (**G**) GST pull-down assays were performed to detect the interactions between OTUD3 and PLK1. After mixing purified GST or GST-labeled OTUD3 with purified Myc-PLK1, pull-down assays were performed using glutathione Sepharose 4B beads. **H** In vitro kinase assays revealed that PLK1 mediates OTUD3 phosphorylation. Recombinant GST-OTUD3 (expressed in *E. coli*) and Myc-PLK1 (expressed in HEK-293T) were collected for in vitro kinase assays. Loading controls are shown by coomassie blue staining. **I** Myc-PLK1 and HA-OTUD3 were transfected into HEK-293T cells. The PLK1 specific inhibitor HMN-214 (3 μM, 6 h) pretreated cells. Co-IP showing OTUD3 phosphorylation in HEK-293T cells. **J** Myc-PLK1 and HA-OTUD3 (wild type) or HA-OTUD3 (S287A, S326A) were transfected into CRC cells. Co-IP and Western-blot revealed the phosphorylation of OTUD3 in CRC cells. **K** The Ser-326 of OTUD3 is conserved in mammals (rat, mouse pig and dog). **L**, **M** Co-IP were performed to confirm the interactions of exogenous PLK1, OTUD3 and YY1 in SW480 cells co-transfected with Myc-PLK1, HA-OTUD3 and Flag-YY1. Anti-HA and anti-Flag antibodies were used for immunoprecipitation, respectively. **N** Co-IP assays were performed to confirm the interactions between OTUD3 and YY1 in HEK-293T cells that had been co-transfected with Myc-PLK1, Flag-YY1 and HA-OTUD3(WT) or HA-OTUD3 (S326A). The binding ability of HA-OTUD3 (S326A) to YY1 was significantly weaker than that of HA-OTUD3 (WT). **O**–**R** Specific plasmids were transfected into SW480 cells, and then treated with cycloheximide (CHX, 50 μg/ml) for the indicated times. Quantification of YY1 levels relative to α-Tubulin are shown. **S** Flag-YY1, His-Ub, HA-OTUD3 with or without Myc-PLK1 were transfected into HEK-293T cells treated with MG132 (10 μM for 6 h). Subsequently, anti-Flag immunoprecipitates of HEK-293T total lysates, and the His-Ub were detected by Western blot. **T** Flag-YY1, His-Ub, HA-OTUD3 or HA-OTUD3 (S326A) were transfected into HEK-293T cells treated with MG132 (10 μM for 6 h). Next, anti-Flag immunoprecipitates of HEK-293T total lysates, and the His-Ub were detected by Western blot.

For accurate biological functions, the activities and functions of DUBs are strictly regulated. Post-translational modifications, especially phosphorylation, play a vital role in regulating DUBs [33]. Akt mediates USP14 phosphorylation at Ser-432 and changes its conformation to promote the binding of USP14 to substrates [34]. As a member of the OTU family, the deubiquitination enzyme activities of OTUD5 are activated when Ser-177 is phosphorylated by CKII kinase and a conformational change occurs near its active site [35]. Therefore, we predicted the potential phosphorylation sites on OTUD3 using the PhosphoSitePlus® PTM database (https://www.phosphosite.org/homeAction.action). The results revealed several potential phosphorylation sites on OTUD3 (Supplementary Fig. S4A). In addition, our data indicated that both exogenous or endogenous OTUD3 were efficiently precipitated and detected by the anti-Ser(P)/Thr(P) antibody (Fig. 5A, B and Supplementary Fig. S4B, C). Next, tandem affinity purification and mass spectrometry were performed to detect the HA-OTUD3 associated protein complex, which identified the serine/threonine protein kinase Polo-like kinase 1 (PLK1) on the prey list (Fig. 5C). Consistent with interactions observed in mass spectrometry analysis, Co-IP assays confirmed the interactions between endogenous OTUD3 and endogenous PLK1 in SW480 and DLD1 cells (Fig. 5D and Supplementary Fig. S4D). Ectopically expressed HA-tagged OTUD3 also interacted with Myc-tagged PLK1 in HEK-293T cells, and vice versa (Fig. 5E and Supplementary Fig. S4E). Immunofluorescent staining indicated that OTUD3 and PLK1 were colocalized in DLD1 and SW480 cells (Fig. 5F). In vitro pull-down assays with purified recombinant proteins showed that Myc-PLK1 directly bound GST-OTUD3, instead of GST control (Fig. 5G).

To explore the regions responsible for the interactions between OTUD3 and PLK1, we generated a set of deletion mutants of PLK1. Each of the PLK1 deletion mutants was co-transfected with OTUD3 (wild-type), or each of the OTUD3 deletion mutants was co-transfected with PLK1 (wild-type) into HEK-293T cells, followed by Co-IP assays. The UBA domain of OTUD3 and the PBD domain of PLK1 were found to be crucial in the interactions between the two proteins (Supplementary Fig. S4F–J). In vitro kinase assays showed that OTUD3 protein was phosphorylated by Myc-PLK1 protein that were purified from HEK-293T cells (Fig. 5H). Then, we further explored the functions of PLK1 in OTUD3 phosphorylation. The expression of phosphorylated form of endogenous OTUD3 in HEK-293T cells and CRC cells was explored. The phosphorylated form of the endogenous OTUD3 protein was elevated by increased expression level of PLK1. Pretreatment with the PLK1-specific inhibitor, HMN-214 (3 μM, 6 h), significantly inhibited OTUD3 phosphorylation (Fig. 5I and Supplementary Fig. S4K–L).

The consensus sequence for PLK1 phosphorylation sites is (E/D/Q)-X-(S/T)-Ø, where X denotes any amino acid and Ø denotes a hydrophobic amino acid. After amino acid sequence analysis of OTUD3, we found that Ser-326 and Ser-287 were consistent with the consensus sequence of PLK1 phosphorylation site. Additional experiments showed that PLK1 failed to phosphorylate OTUD3 with Ser-326A mutations (Fig. 5J). Moreover, we found that Ser-326 was conserved among mammals (rat, mouse pig and dogs) (Fig. 5K). Then, the immunoprecipitation assays confirmed that up-regulated PLK1 promoted the physical binding between OTUD3 and YY1, but failed to promote the binding between OTUD3 mutation (S326A) and YY1 (Fig. 5L–N and Supplementary Fig. S5A, B). Treatment with the PLK1 inhibitor, HMN-214, significantly inhibited the binding between OTUD3 and YY1 (Supplementary Fig. S5C, D). Further, results showed that under cycloheximide (CHX) treatment, the OTUD3 phosphorylated defective mutant (S326A) also failed to prolong the half-life of YY1, compared to OTUD3 (wild-type) (Fig. 5O, P and Supplementary Fig. S5E, F). Over-expressed PLK1 enhanced the effects of OTUD3 on extending the half-life of YY1. However, pretreatment with HMN-214 significantly shortened the half-life of YY1 (Fig. 5Q, R and Supplementary Fig. S5G, H). Moreover, consistent with these findings, up-regulation of PLK1 promoted the deubiquitination of YY1 by OTUD3 (wild-type), while OTUD3-S326A weakened the deubiquitination of YY1, compared to OTUD3(wild-type) (Fig. 5S, T). In summary, phosphorylation of the OTUD3 protein enhances its ability to bind YY1, ultimately further maintaining YY1 protein stability.

### Elevated OTUD3 expression positively correlates with YY1 up-regulation and predicts poor prognostic outcomes for CRC patients

**Fig. 6:**
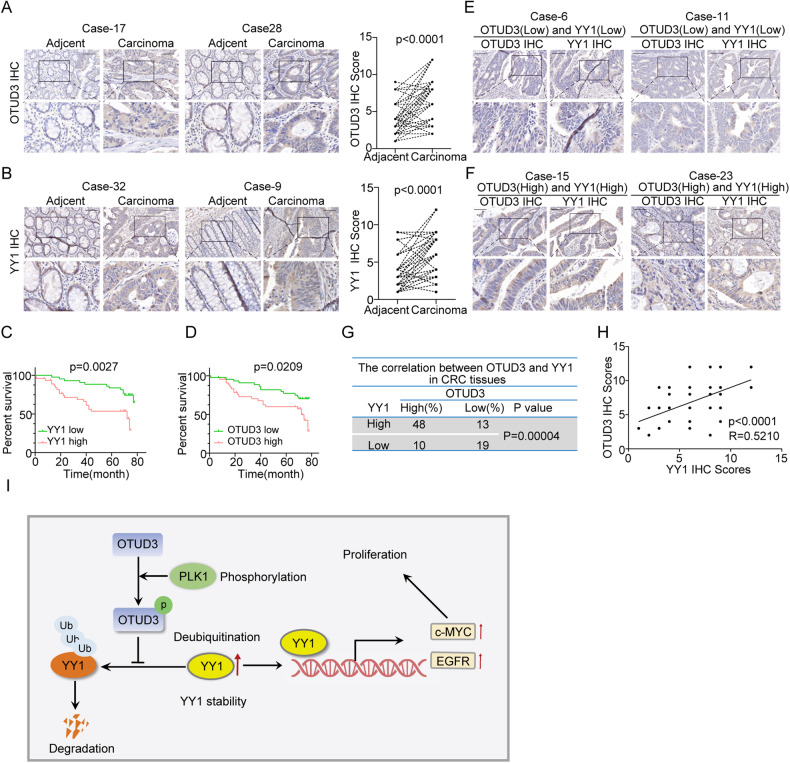
Up-regulated OTUD3 is positively correlated with YY1 and predicts poor prognostic outcomes in CRC. **A**, **B** The IHC assays were performed to detect OTUD3 and YY1 expression levels in CRC (*n* = 90) and their corresponding adjacent tissues (*n* = 90). Paired T-tests were used to analyze the variations between CRC and the corresponding para-cancerous tissues. Scale bar, 100 μm. **C** Stratified by OTUD3 protein levels, a Kaplan–Meier curve was drawn for overall survival of CRC patients, and statistical differences were analyzed by log-rank test. **D** Stratified by YY1 protein levels, a Kaplan–Meier curve was drawn for overall survival outcomes of CRC patients, and statistical differences were analyzed by log-rank test. **E**–**G** A positive correlation between OTUD3 and YY1 protein levels in CRC. Statistical differences were analyzed by Chi-square test and r represents Spearman’s correlation coefficient. Scale bar, 100 μm. **H** In individual CRC patients, the YY1 protein level score (X-axis) was positively correlated with OTUD3 protein level score (Y-axis). Statistical differences were analyzed by linear regression and r is the correlation coefficient. **I** Model showing that OTUD3 phosphorylation promotes YY1 deubiquitination and promotes CRC proliferation.

Numerous studies have shown that up-regulation of YY1 protein in CRC is correlated with CRC progression. In this study, we found that OTUD3 can promote YY1 protein deubiquitination and stabilization. Therefore, we postulated that OTUD3 promotes CRC progression by up-regulating YY1. To investigate this hypothesis, immunohistochemical (IHC) staining was performed to assess the expression of OTUD3 and YY1 in CRC tissues (*n* = 90) and adjacent tissues (*n* = 90). The OTUD3 and YY1 were highly expressed in CRC tissues, compared to matched adjacent tissues (Fig. 6A, B). Compared to CRC patients with suppressed OTUD3 or YY1 expression, those with elevated levels of these proteins were associated with significantly shorter overall survival outcomes (Fig. 6C, D). The OTUD3 and YY1 protein expression levels were positively correlated in the tumor samples (Fig. 6E–H). Overall, these findings are in agreement with our experimental findings on OTUD3-mediated YY1 stability.

## Discussion

YY1 is a zinc finger protein that positively or negatively regulates the transcription of numerous genes in different cellular processes [6, 7]. Expression of YY1 has been associated with emergence of drug resistance, cancer metastasis and poor prognostic outcomes. Although YY1 plays important roles in cancer progression, the mechanisms by which YY1 promotes tumor growth are complex and not well understood. As a transcription factor or inhibitor, YY1 has two roles in cancer: tumor promotion or tumor suppression [36]. The contrasting roles of YY1 as a tumor suppressor or tumor promoter likely rely on its interacting partners in a different tumor setting, which suppresses or promotes the expression of several genes and non-coding RNAs. For instance, YY1 inhibition of miR-29, miR-9, miR-489 and miR-146a is pro-tumorigenic [37–40]. However, YY1 mitigates pancreatic ductal adenocarcinoma metastasis by suppressing MMP10 transcription [41].

CRC is a major health concern and the significance of YY1 in accelerating tumor progression has been reported [42]. YY1, a transcription factor, is overexpressed in CRC and promotes tumor growth, metastasis and drug resistance [9, 12]. In CRC, YY1 combines with the promoter of miR-500a-5p, thereby suppressing its transcription and weakening the inhibition of HDAC2 by miR-500a-5p, leading to promotion of CRC proliferation [15]. Additionally, YY1 promotes colon cancer progression by promoting the Wnt signaling pathway and inhibiting p53 [43]. Consistent with these findings, we found that YY1 was abnormally highly expressed in CRC tissues, and promoted CRC proliferation in vitro and in vivo. Elevated YY1 expression was strongly associated with shorter survival outcomes, however, the mechanism of YY1 high expression in CRC remains unknown.

Previous studies have shown that YY1 stability is regulated by ubiquitination, and that YY1 is a substrate for deubiquitination enzymes. In this study, we established that OTUD3 is a novel DUB of YY1. OTUD3 enhances the stability of YY1 by cleaving ubiquitin molecules from YY1 at K332, K339 and K409 residues. Our findings suggest that OTUD3 promotes CRC progression by up-regulating YY1, and provides insights into the underlying mechanisms of YY1 over-expression in CRC. Further studies on regulation of YY1 stability by DUBs, such as OTUD3 may provide new idea for the diagnosis and treatment of CRC.

DUBs have been extensively studied due to their potential role in stabilizing oncoprotein during cancer progression. As a member of DUBs, OTUD3 remove ubiquitin from its substrate, and its role in tumor progression depends on specific substrates. OTUD3 promotes lung tumorigenesis by stabilizing GRP78 [44], while it stabilizes ACTN4 to drive hepatocellular carcinoma progression and metastasis [45]. In breast cancer, OTUD3 suppresses tumorigenesis and progression by stabilizing PTEN [46]. We found that OTUD3 directly interacts with YY1. Over-expression of OTUD3 significantly increased YY1 protein expression and suppressed its ubiquitination, whereas OTUD3 knockdown or knockout increased YY1 degradation. Furthermore, OTUD3 stabilized YY1 by removing K48-linked polyubiquitination. We confirmed that K332, K339 and K409 loci, which are located in the ZNF domain of YY1, are important for OTUD3 mediated deubiquitination. Importantly, we found that OTUD3 acts as an oncogene to accelerate CRC tumor growth by enhancing YY1 stability while YY1 depletion reversed OTUD3 overexpression-induced tumor progression. Compared with adjacent normal tissues, OTUD3 expression level was significantly increased in CRC tissues, which may be one of the reasons why YY1 protein levels were highly expressed in CRC.

Numerous studies have demonstrated that phosphorylation affects the functions of DUBs [47]. We found that OTUD3 was also phosphorylated. Mass spectrometry identified PLK1 to be an interacting partner of OTUD3. In TGF-β-induced EMT, PLK1 binds and phosphorylates Ser-311 of β-catenin to facilitate non-small cell lung cancer metastasis [48]. PLK1 and AURKB mediated survivin phosphorylation promotes triple-negative breast cancer proliferation [49]. In this study, we found that the PBD domain of PLK1 binds OTUD3. Moreover, PLK1 phosphorylates Ser-326 of OTUD3, thereby promoting the binding and stabilization of YY1. However, the underlying mechanisms by which phosphorylation enhances OTUD3-mediated deubiquitination of YY1 protein have yet to be elucidated, and we hope to explore this in the future.

In summary, our study suggests a unique role of OTUD3 in controlling CRC tumor growth. Therefore, the OTUD3-YY1 signaling pathway is a potential treatment target for CRC. Identification of OTUD3 phosphorylation by PLK1 adds a new layer of complexity to regulation of this signaling pathway and highlights the importance of phosphorylation in regulation of DUBs (Fig. 6I). Our findings will provide new ideas for developing new therapeutic options for CRC.

## Materials and methods

### Cell culture

Human cell lines (SW480, SW620, HCT116, HT29, DLD1, LOVO, and HEK-293T) were obtained from the American Type Culture Collection (ATCC) and maintained under ATCC-recommended culture conditions. The reagents utilized in this study are shown in the Supplementary Material 3.

### GST pull-down assay

GST-OTUD3 was purified from lysates of *Escherichia coli* BL21 (DE3) over-expressing GST-OTUD3 recombinant proteins using Glutathione Sepharose 4B beads (Amersham Pharmacia Biotech). Flag-YY1 or Myc-PLK1 were transfected into HEK-293T cells and incubated overnight at 4 °C with the GST protein or GST-OTUD3 protein. Glutathione-sepharose 4B beads pull down the GST-OTUD3 complex. Bound fusion proteins were isolated from beads and examined by immunoblotting with anti-Flag or anti-Myc antibodies (The buffer of GST pull-down assay is presented in the Supplementary Material 3).

### Proximity ligation assay (PLA)

CRC cells were fixed in 4% paraformaldehyde for 10 min, blocked with 5% BSA for 1 h and incubated overnight at 4 °C with the corresponding antibodies. The proximity ligation assay (PLA) was performed using the Duolink In Situ PLA kit (Sigma-Aldrich), according to the manufacturer’s instructions. DAPI was used to localize the nuclei after which images were taken using a confocal microscope.

### Immunohistochemical (IHC) staining

CRC TMAs were obtained from Shanghai Ouduo Biotechnology Co., Ltd. There were 90 pairs of cancerous and surrounding normal tissues. The IHC assay was performed as previously reported [50]. Immunohistochemical scoring was based on: staining intensity was scored as 0 points (none), 1 point (weak), 2 points (moderate), 3 points (strong); staining ratio was scored as 1 point (25%), 2 points (25–50%), 3 points (50–75%), and 4 points (>75%). Then, IHC staining was separately scored by two pathologists. Final IHC scores (H-Scores) were calculated from the intensity score of stained cells multiplied by the staining ratio score (0-12). Low and high expression levels of OTUD3 or YY1 were H-score <6 and ≥6, respectively.

### Immunofluorescence (IF) staining

Immunofluorescence staining was performed as previously reported [44].

### Western blotting (WB)

This assay was performed as previously described [50]. Primary antibodies specific for the following proteins were used at a dilution ratio of 1:1000.

### Plasmid and siRNA transfection and lentiviral transduction

The human DUB expression plasmids used in this study were purchased from OriGene (Maryland, USA). Full-length OTUD3-WT, OTUD3-C76A, OTUD3-S326A, OTUD3-S287A, Full-length YY1-WT and YY1 mutants (4KR, K332, K339, K393 and K409) were cloned into the pCDNA3.1-CMV-HA-C or pCDNA3.1-CMV-Flag-C vectors as indicated. GST-tagged OTUD3 was cloned into the pGEX-6P-1 vector. YY1 mutants (ΔN, Δ200-257, ΔZNF) were cloned into the pCDNA3.1-CMV-Flag-C vector. HA-OTUD3 mutants (ΔOTU, ΔUBA, ΔTail) were cloned into pCDNA3.1-CMV-HA-C vector. PLK1 and PLK1 mutants (ΔKIN and ΔPBD) were cloned into the pCDNA3.1-CMV-Myc-C. Ub-WT and Ub mutants (K6, K11, K27, K29, K33, K48, K48R, K63, K63R) were cloned into the pCDNA3.1-CMV-His-C vector. These plasmids were transfected into cells using the Lipofectamine 3000 transfection reagent (Thermo Fisher Scientific, USA), as instructed by the manufacturer.

To stably down-regulate OTUD3 or YY1 expression, GV248 lentiviral vectors were used to express OTUD3 or YY1 shRNA and puromycin sequences. To generate cell lines with stable OTUD3 or YY1 down-regulation, CRC cells were transfected with lentiviral vectors expressing OTUD3 sh-RNA or YY1 sh-RNA (MOI = 10), while puromycin (2 μg/ml, InvivoGen) was added 48 h after infection to isolate stable cell clones. The shRNA details are provided in Supplementary Table S1.

### CRISPR-Cas9

The OTUD3 gene was knocked out using the CRISPR/Cas9 technology (GenePharma Co., Ltd. Shanghai, China). Specifically, sgRNA GAGGATCAATGAC AACTCAG was designed to target the coding region of the OTUD3 gene, and was subsequently cloned into the lentiCRISPRv1 vector. The virus was harvested from HEK-293T cells and tranfected into SW480 cells. Puromycin was utilized to select cells with successful OTUD3 knockout. Single cell clones were obtained via limiting dilution, and OTUD3 deletion was confirmed by both qPCR and Western blotting.

### Colony formation assay

The colony formation assays were performed as previously reported [44].

### Cell proliferation assay

The cell proliferation assays were performed as previously reported [44].

### Subcutaneous xenograft implantation models

To establish subcutaneous xenograft implantation models, CRC cells (10 × 10^6^) were subcutaneously implanted into 6-week-old male nude mice. All animals were euthanized after 28 days. Tumor volumes were measured after every 3 days.

### In vivo ubiquitination assay

The corresponding plasmids were transiently transfected into HEK-293T cells. The ubiquitination assay was performed as previously reported [51].

### Statistical analysis

Statistical analyses were performed using SPSS 20.0 and GraphPad Prism 06 softwares. All quantitative data, including relative expression data obtained from Western blot analysis, colony formation assays, and cell proliferation assays, are presented as mean ± standard deviation (SD) from at least three independent experiments. Comparisons of means between groups was performed by a two-tailed unpaired or paired Student’s *t* test. Correlations between OTUD3 and YY1 were determined using the chi-square test and linear regression analysis. Kaplan–Meier survival curves were assessed using the log-rank test. Statistical significance was set at *p* ≤ 0.05 (*p < 0.05, **p < 0.01, ***p < 0.001).

### In vitro kinase assay

To assess the phosphorylation of OTUD3 by PLK1, recombinant GST-OTUD3 (expressed in *E. coli*) and Myc-PLK1 (expressed in HEK-293T) were mixed in 40 μl kinase assay buffer (50 mM glycerol phosphate, 10 mM HEPES (pH 7.5), 10 MgCl_2_, 50 mM NaCl, 30 μM ATP, 1 mM DTT, 10 mM MnCl_2_) and incubated at 30 °C for 30 min. Next, they were incubated at 4 °C for 8 h in the presence of GST antibody-coupled magnetic beads. The phosphorylation levels of GST fusion protein were analyzed by Western blotting.

### Immunoprecipitation assay

A protease inhibitor cocktail was added into pre-chilled IP buffer(50 mM Tris-Hcl pH7.4, 1% NP-40, 150 mM NaCl, 0.05 mM EDTA, 10% Gly) and the cells lysed. Whole cell extracts were collected and incubated overnight with 1 μg of the corresponding antibody and A/G magnetic beads or labeled antibody-conjugated magnetic beads. After washing the beads five times, they were boiled with the IP buffer for SDS-PAGE analysis.

### LC-MS/MS

The HA-OTUD3 or Flag-YY1 were transfected into DLD1 cells. Anti-HA or anti-Flag affinity magnetic beads were used for immunoprecipitation. The proteins were identified by mass spectrometry (Spec-ally (Wuhan) Life Technology Co., Ltd.).

### Ubiquitination proteomics

The DLD1 cells were transfected with HA-OTUD3 after which 4D ubiquitination proteomics was performed at Hangzhou Jingjie Biological (PTM) Laboratory Co., Ltd. (China).

### Supplementary information

Figure.S1

Figure.S2

Figure.S3

Figure.S4

Figure.S5

Supplemental Material1

Supplemental Material2

Supplemental Material3

Original Data File

## Data Availability

The data that support the findings of this study—including Ubiquitination proteomics data and LC-MS/MS. The data of ubiquitination proteomics were uploaded to ProteomeXchange Consortium (https://proteomecentral.proteomexchange.org/cgi/GetDataset?ID=PXD042000). The data of LC-MS/MS are provided in the paper and its Supplementary Information.
