# Supplementary figures and images for: Phosphorylation-dependent deubiquitinase OTUD3 regulates YY1 stability and promotes colorectal cancer progression
Source: Cell Death Dis. 2024 Feb 13;15(2):137. doi: 10.1038/s41419-024-06526-8 (PMC10864350; doi:10.1038/s41419-024-06526-8)

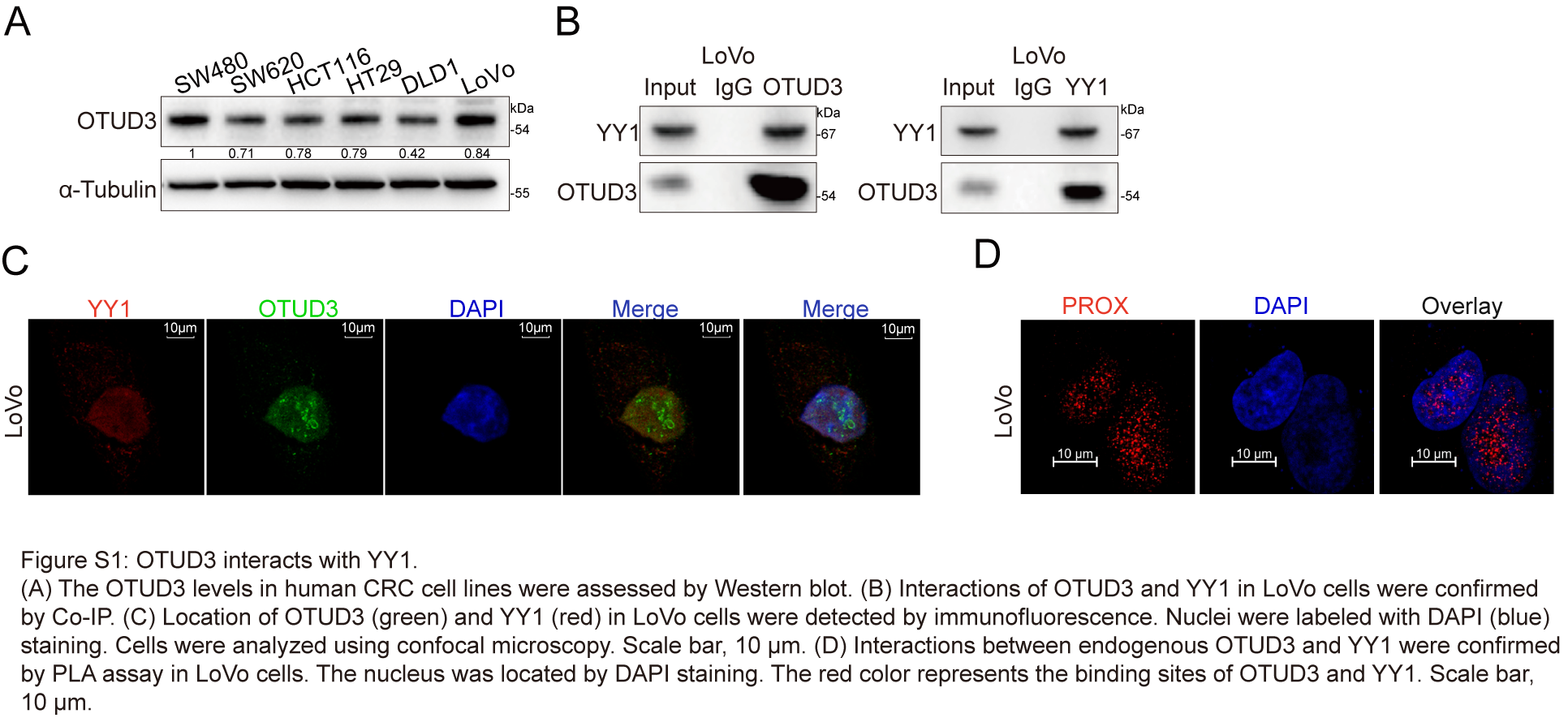

Supplement: Supplementary file 1 — Figure.S1 [file 41419_2024_6526_MOESM1_ESM.tif]

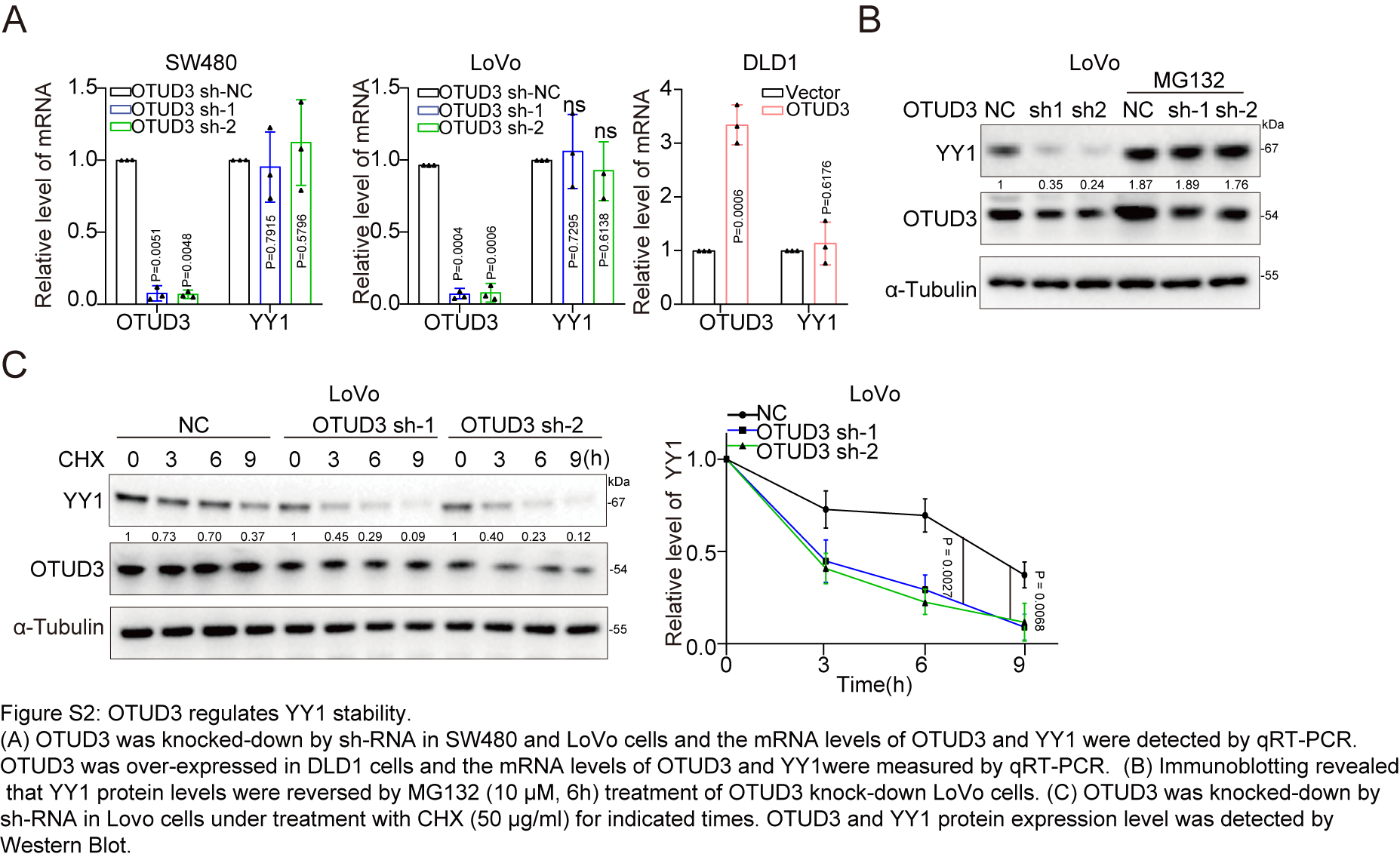

Supplement: Supplementary file 2 — Figure.S2 [file 41419_2024_6526_MOESM2_ESM.tif]

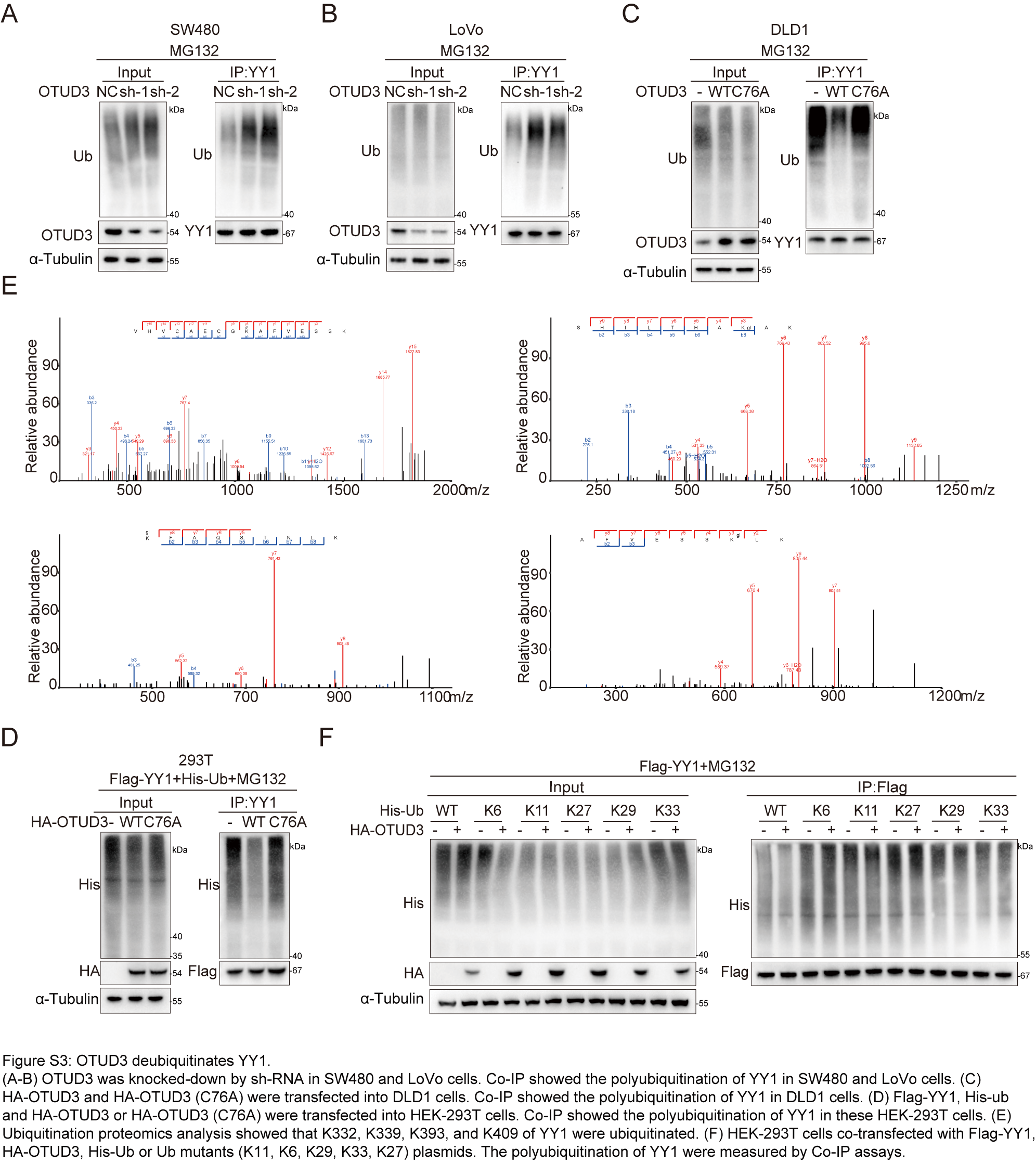

Supplement: Supplementary file 3 — Figure.S3 [file 41419_2024_6526_MOESM3_ESM.tif]

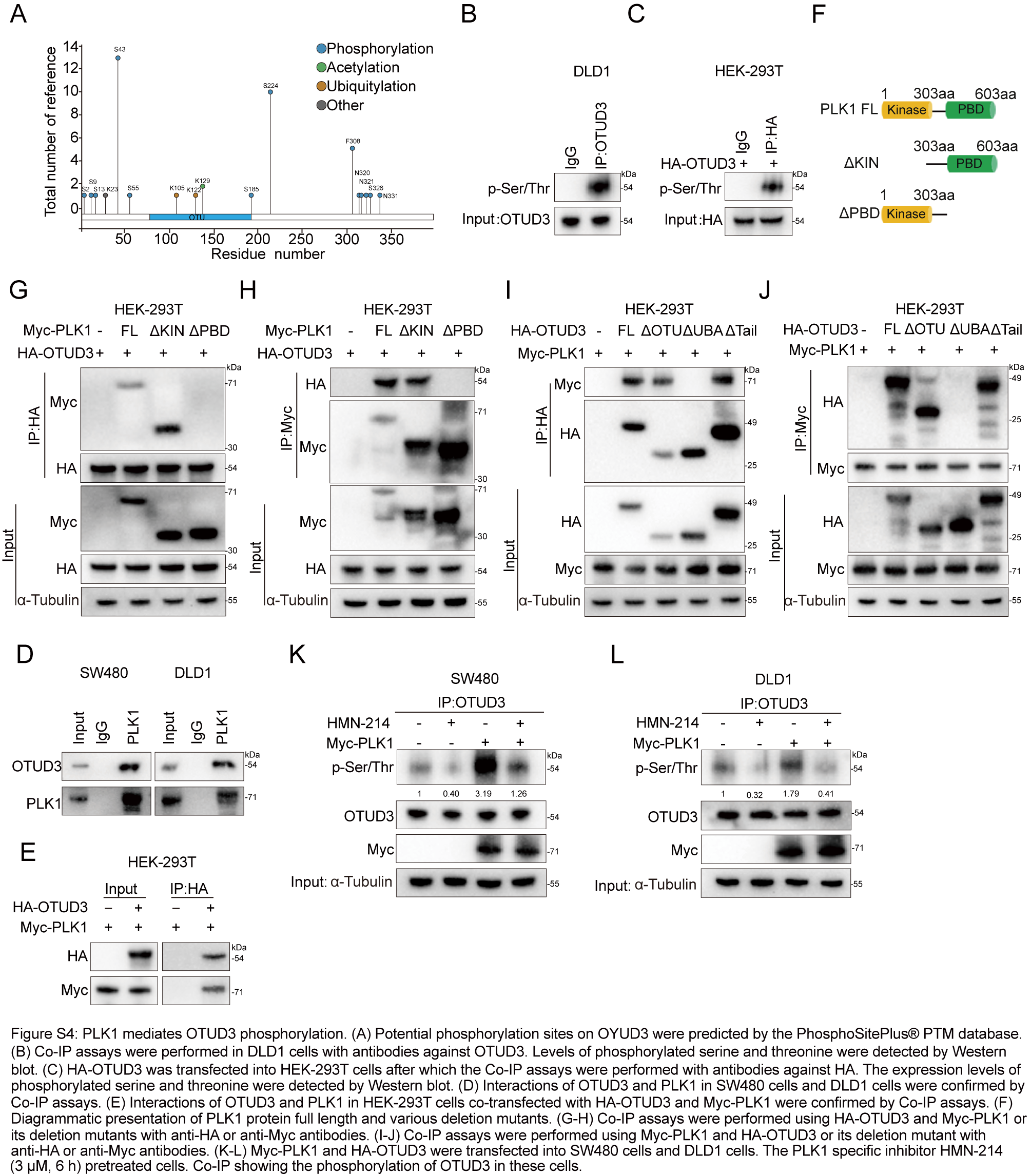

Supplement: Supplementary file 4 — Figure.S4 [file 41419_2024_6526_MOESM4_ESM.tif]

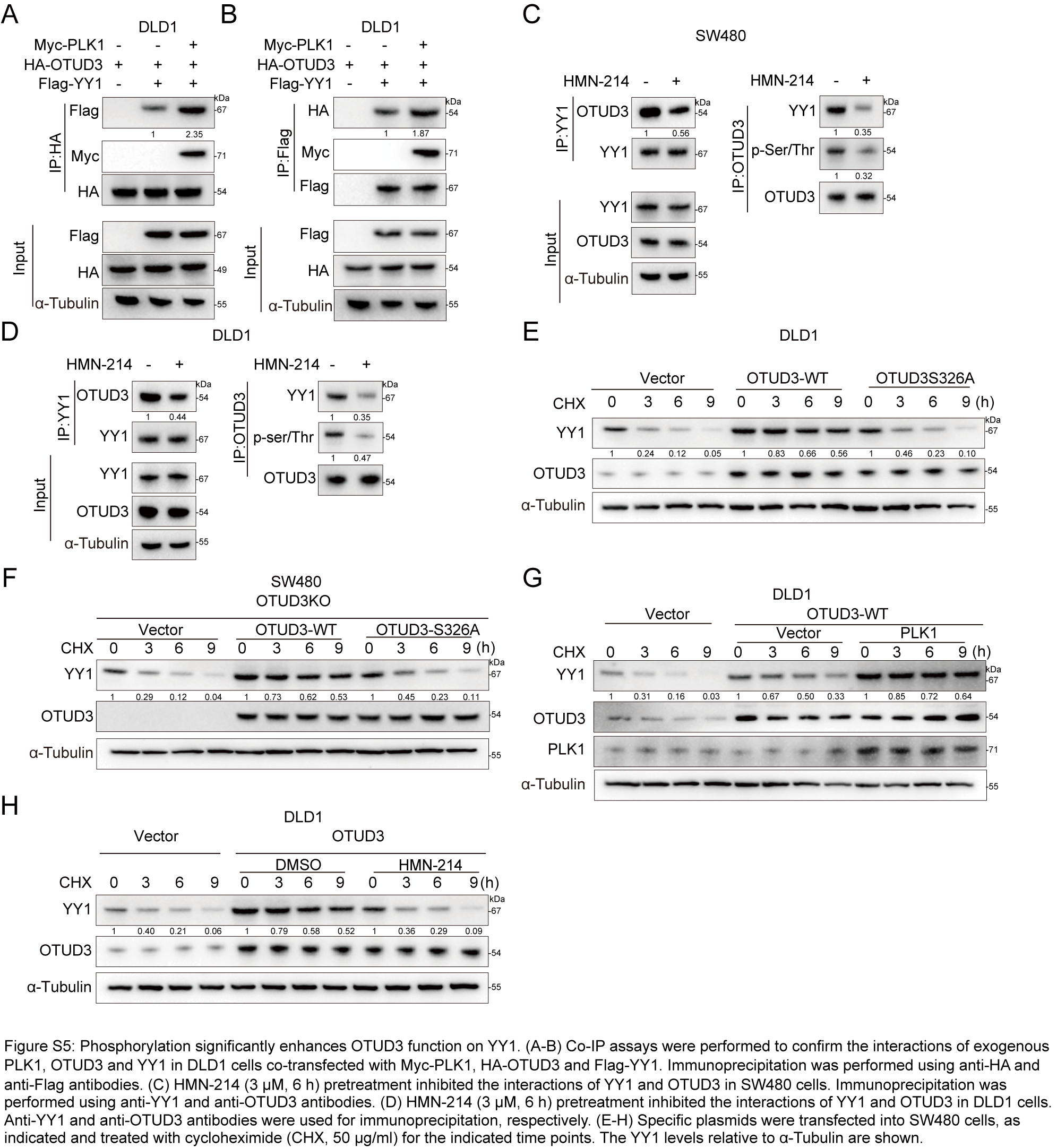

Supplement: Supplementary file 5 — Figure.S5 [file 41419_2024_6526_MOESM5_ESM.tif]

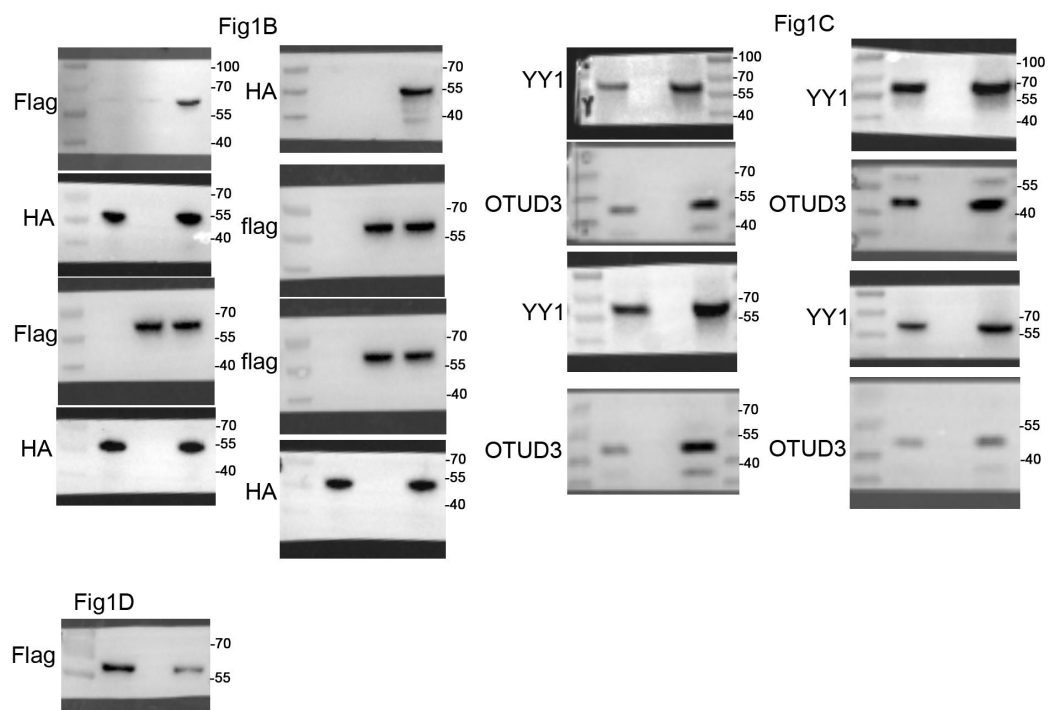

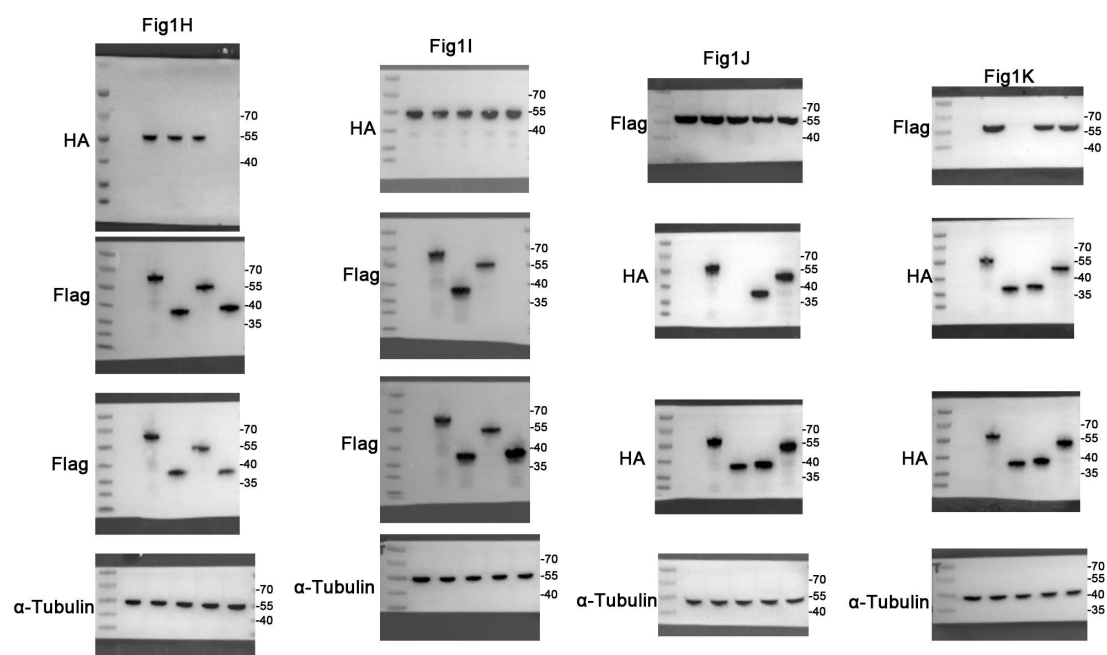

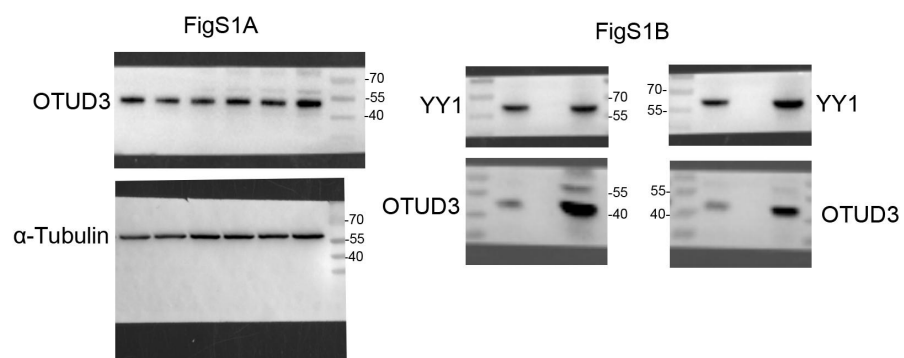

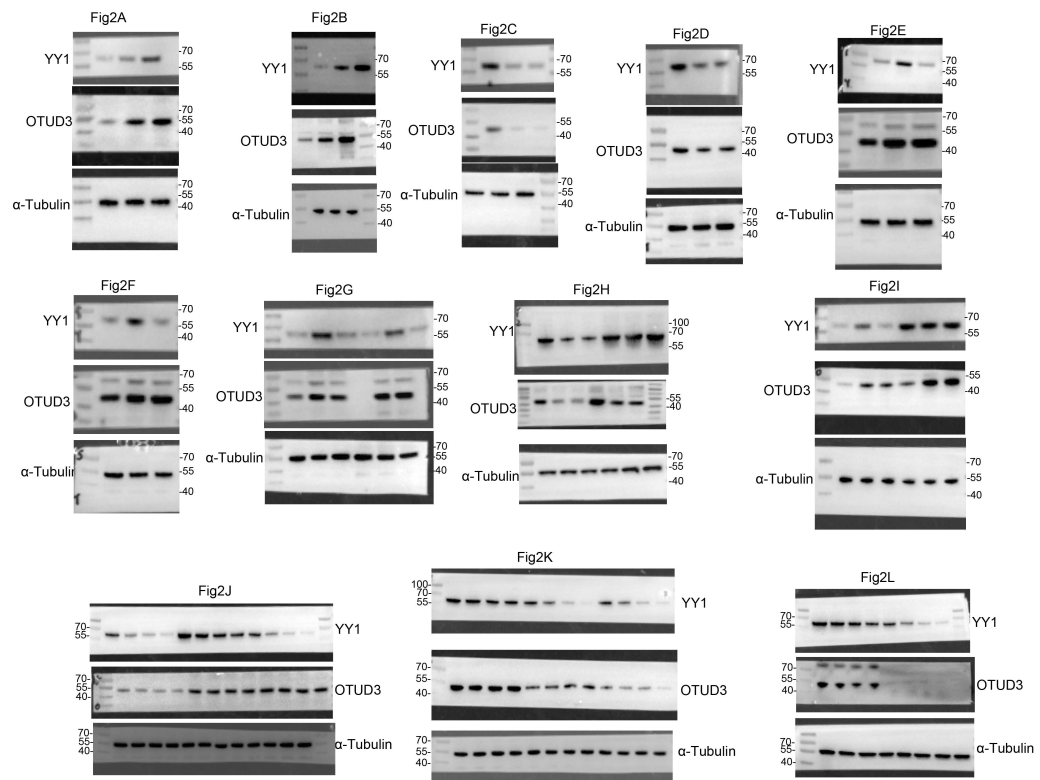

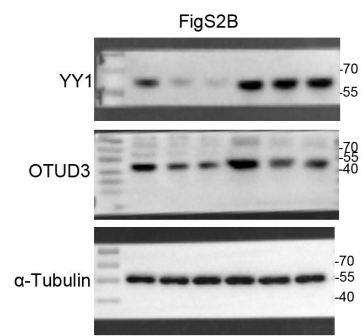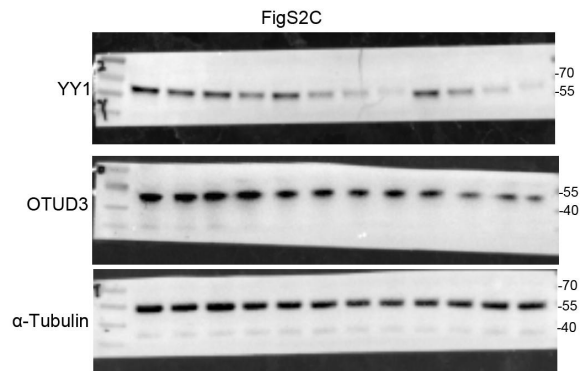

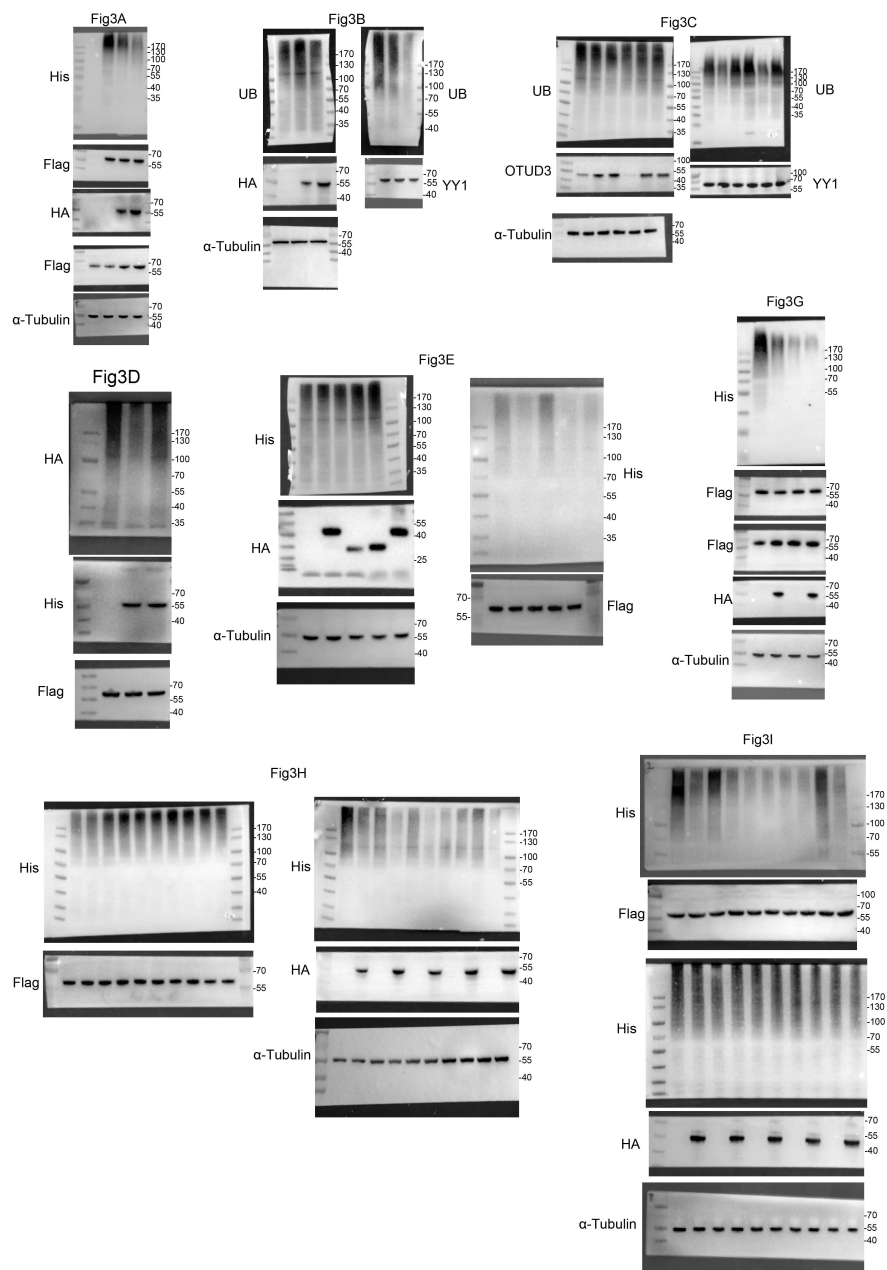

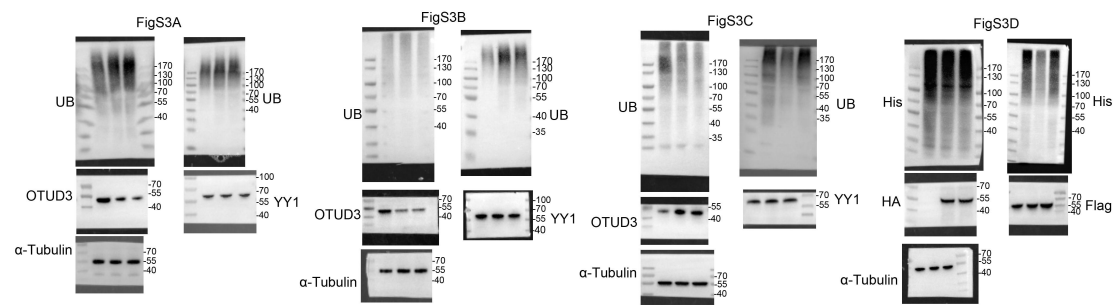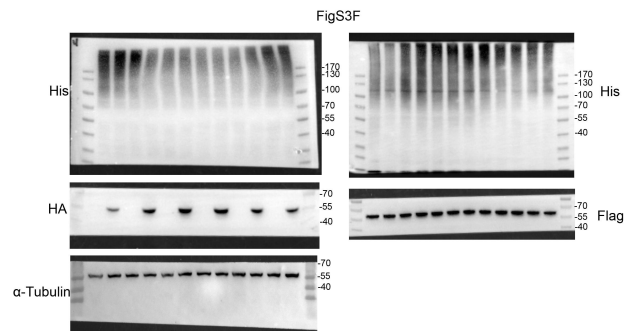

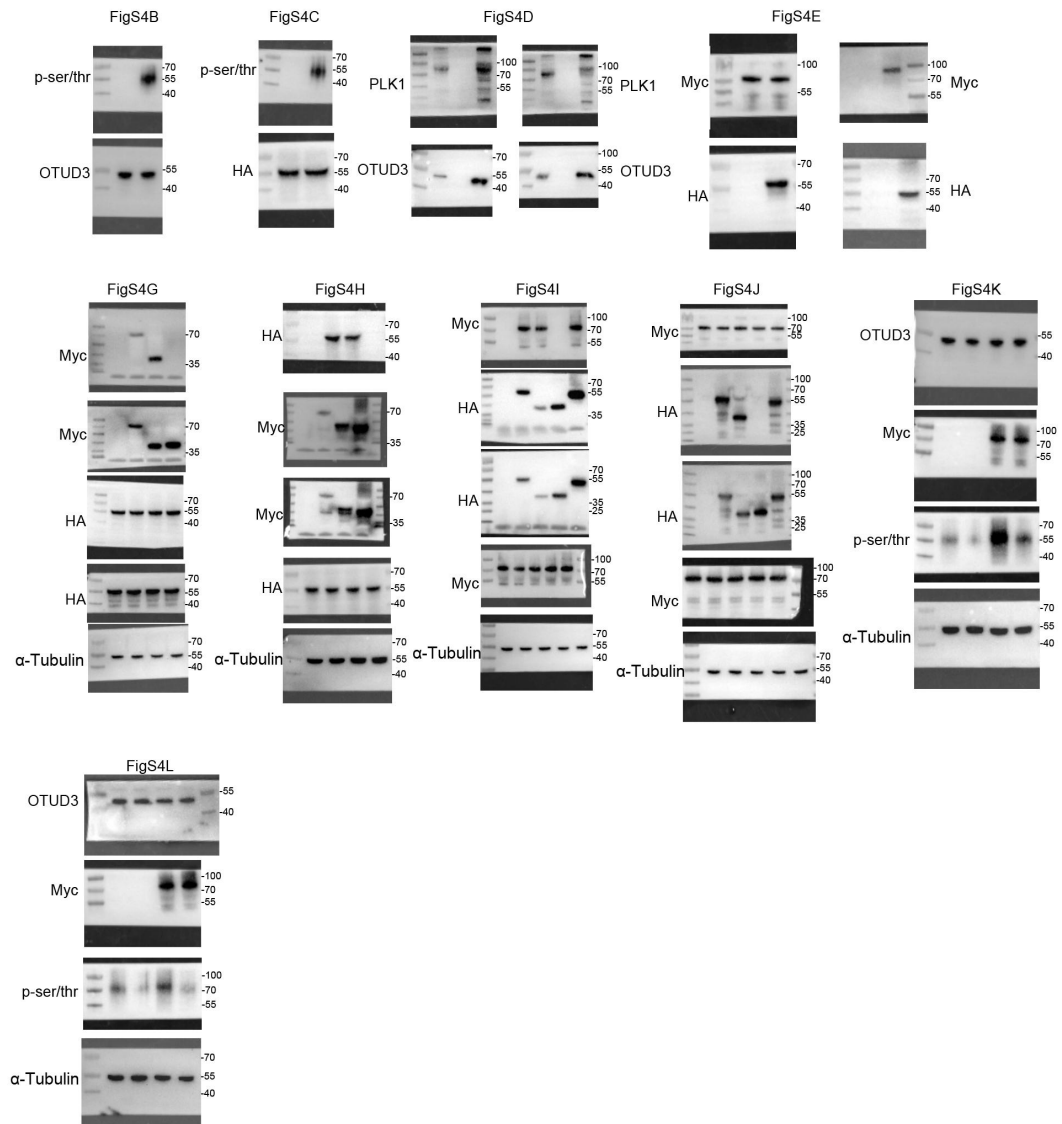

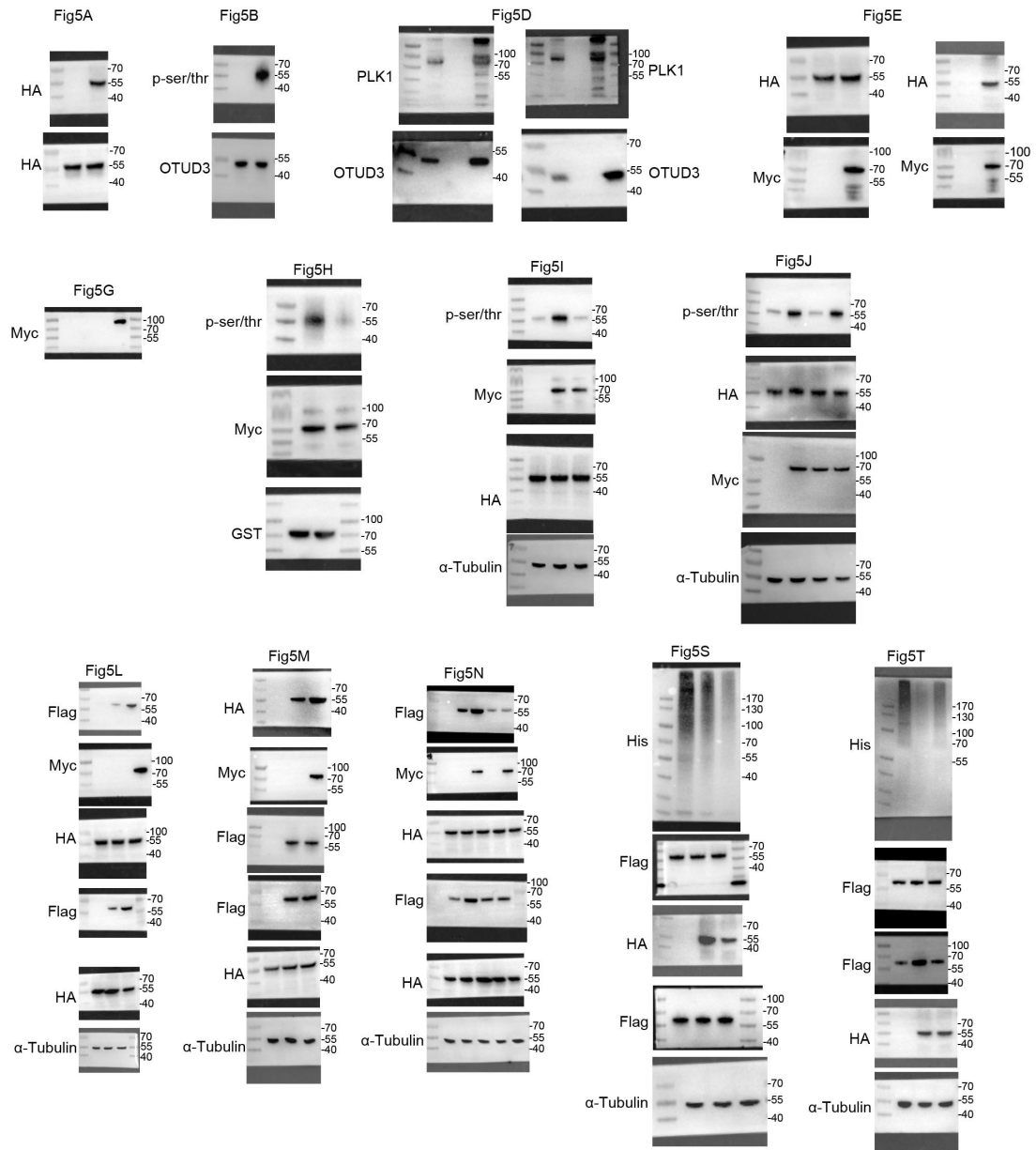

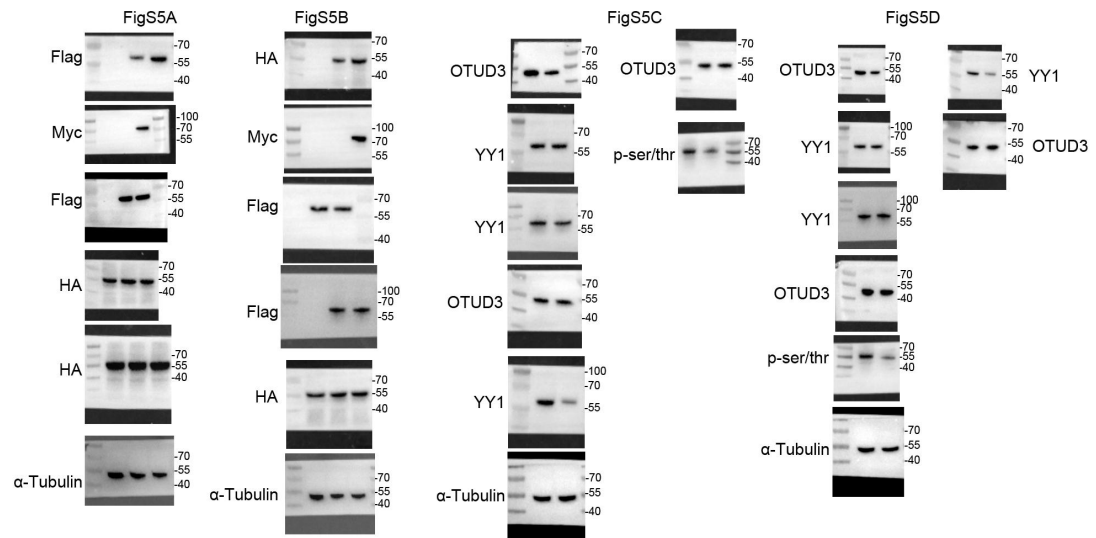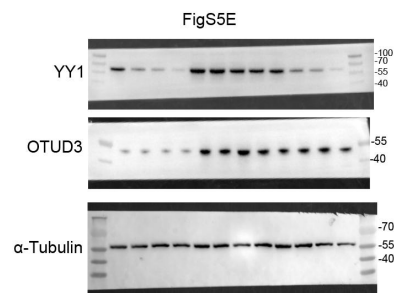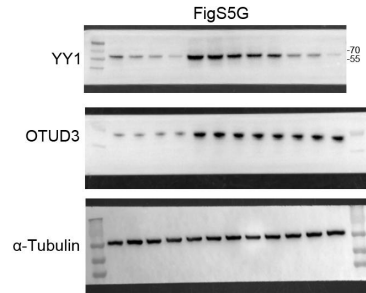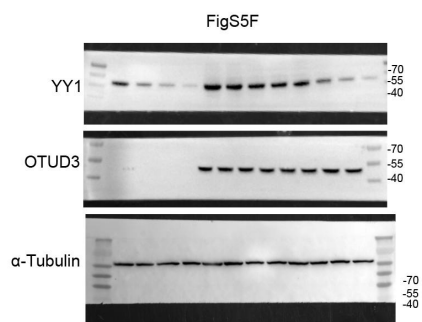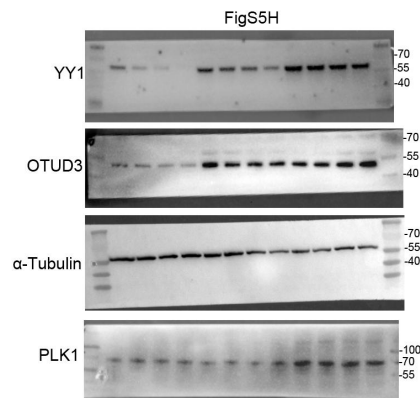

Supplement: Supplementary file 9 — Original Data File [file 41419_2024_6526_MOESM9_ESM.pdf]
