# Supplementary material for: Phosphorylation-dependent deubiquitinase OTUD3 regulates YY1 stability and promotes colorectal cancer progression
Source: Cell Death Dis. 2024 Feb 13;15(2):137. doi: 10.1038/s41419-024-06526-8 (PMC10864350; doi:10.1038/s41419-024-06526-8)
Supplement: Supplementary file 8 — Supplemental Material3 [file 41419_2024_6526_MOESM8_ESM.docx]

Table S1: The antibodies and reagents used in this study.

| REAGENT or RESOURCE | RESOURCE | IDENTIFIER |
| --- | --- | --- |
| **Antibodies** | | |
| OTUD3 | Proteintech | 29622-1-AP |
| YY1 | Proteintech | 22156-1-AP |
| α-Tubulin | Proteintech | 66031-1-Ig |
| HA | Cell Signaling Technology | #3724 |
| Flag | Cell Signaling Technology | #14793 |
| Myc | Cell Signaling Technology | #2276 |
| His | Cell Signaling Technology | #12698 |
| PLK1 | Cell Signaling Technology | #4513 |
| ubiquitin | Santa Cruz Biotechnology | sc-8017 |
| Phospho-(Ser/Thr) Antibody | Cell Signaling Technology | #9631 |
| **Chemicals** | | |
| MG132 | MCE | [HY-13259](https://www.medchemexpress.cn/MG-132.html) |
| Cycloheximide (CHX) | MCE | [HY-12320](https://www.medchemexpress.cn/Cycloheximide.html) |
| Cyclapolin 9 | MCE | HY-15159 |
| PMSF | MCE | [HY-B0496](https://www.medchemexpress.cn/pmsf.html) |
| **Recombinant DNA** | | |
| OTUD3-shRNA1 | 5′-TGGAAATCAGGGCTTAAAT-3′ |  |
| OTUD3-shRNA2 | 5′-GAAATCAGGGCUUAAATGA-3′ |  |
| YY1-shRNA1 | 5′- GAACTCACCTCCTGATTAT-3′ |  |
| YY1-shRNA2 | 5′-TGCAGATGCTTTCTCATAGCAGAGT-3′ |  |
| OTUD3-sg1： | 5′-GAGGATCAATGACAACTCAG-3′ |  |
| OTUD3-sg2： | 5′-TGGACTACATGATAAAGCAG-3′ |  |
|  |  |  |

Table S2: The buffer was used in GST-pulldown assay.

| Lysis buffer | 50 mM Tris-Cl (pH 7.4), 150 mM NaCl, 1% Triton X-100, 1% sodium deoxycholate, 0.1% SDS和1mM PMSF |
| --- | --- |
| Reduced Glutathione Buffer | 50mM Tris-Cl, 150mM NaCl, 10mM GSH, pH8.0 |
| Binding buffer | 50mM Tris-HCl, 200mM NaCl, 1mM EDTA, 1%(v/v) NP-40, 1mM DTT, 10mM MgCl2, 1xinhibitor Cocktail, pH 8.0 |
| Elution buffer | 50mM Tris-HCl, 400mM NaCl, 50mM GSH, 1mM EDTA, 1mM DTT, 1xinhibitor Cocktail, pH 8.0 |

***In vitro* deubiquitination assay**

*In vitro* deubiquitination assay was performed as described previously. First, Flag-YY1 and HA-Ub expression vectors were co-transfected into HEK293T cells for 24 h. Next, cells were treated with 10 μM MG132 for 6 h, followed by immunoprecipitation with Flag affinity beads to extract the ubiquitinated YY1 protein. Subsequently, the immunoprecipitates were washed three times with the ubiquitination wash buffer followed by elution in BC100 buffer using 3×Flag peptide (F4799, Sigma-Aldrich). His-OTUD3 and His-OTUD3(C76A) were expressed and purified from E. coli, respectively. The ubiquitinated YY1 protein was subsequently incubated with 200 ng of recombinant OTUD3 protein or His-OTUD3(C76A) tag in deubiquitination buffer (50 mmol/L Tris-HCl pH 8.0, 50 mmol/L NaCl, 1 mmol/L EDTA, 10 mmol/L DTT and 5% glycerol) for 2 h at 37 °C, respectively, and YY1 ubiquitination level was analyzed by Western blot.
